# Assessment of collagen hemostatic membrane and sponge in alveolar sockets: A quasi-experimental prospective clinical study

**DOI:** 10.4317/medoral.27642

**Published:** 2025-10-17

**Authors:** Leandro Lécio-de-Lima Sousa, Flávia Priscila Pereira Faco, Luiz Guilherme Fiorin, Tarcio Hiroshi Ishimine Skiba, Juliana Campos Hasse Fernandes, Gustavo Vicentis Oliveira Fernandes, Sergio Charifker Ribeiro Martins, Helio Doyle Pereira-da-Silva

**Affiliations:** 1 Department of Periodontology, Dental Research Division, Guarulhos University, Guarulhos, SP 07023-070, Brazil; 2 Centro Universitário de Santa Fe do Sul (UNIFUNEC), SP, Brazil; 3 Department of Diagnosis and Surgery, Division of Periodontics, São Paulo State University “Júlio de Mesquita Filho” (UN-ESP), Araçatuba, SP, Brazil; 4 Private Researcher, St. Louis, MO, USA; 5 A. T. Still University - Missouri School of Dentistry & Oral Health, St. Louis, MO, USA

## Abstract

**Background:**

This prospective split-mouth clinical study aimed to compare and evaluate bleeding, soft tissue healing, and pain management following tooth extractions using either the Lumina Coat® membrane or the Hemospon® sponge.

**Material and Methods:**

Thirty-two alveoli were included in this study. Following extraction, the sockets were filled with one of two materials: the Hemospon® collagen sponge (control group) or the Lumina Coat® collagen membrane (test group). Participants were assessed at 30 minutes, 24 hours and 48 hours postoperatively to evaluate bleeding, and were then recalled after 7 days for clinical assessment of soft tissue healing and suture removal. Soft tissue healing and bleeding were scored on a 0-3 scale, while pain was assessed using the visual analog scale (VAS). Data were expressed as means with standard deviations and analyzed using Prism 9 (GraphPad Software).

**Results:**

During the later postoperative period, pain scores decreased in both groups. Initial pain scores were 2.20 in the Lumina Coat group and 2.80 in the Hemospon group. By day 7, both groups reported an average score of 0.25 (p>0.05). Bleeding scores were elevated in both groups in the early postoperative phase. The Lumina Coat group had an average score of 1.35, compared with 1.65 in the Hemospon group. Scores decreased progressively over the first 24 hours, after which bleeding resolved completely. A statistically significant difference was observed at 30 minutes, with the Lumina Coat group showing lower scores (average 0.75) compared with the Hemospon group (average 1.20). Healing scores followed a similar pattern - higher in the immediate postoperative period but declining after 7 days. At 2 days postoperatively, the Lumina Coat group demonstrated significantly lower healing scores (average 0.35) than the Hemospon group (average 0.80).

**Conclusions:**

Both materials were effective in controlling pain, reducing bleeding, and promoting soft tissue healing. However, the Lumina Coat group showed superior outcomes compared to Hemospon, particularly in reducing bleeding and improving healing scores.

Keywords: Extraction, socket, hemostasis, bleeding, membrane, sponge, collagen.

## Introduction

Tooth extractions are among the most common surgical procedures performed by dental surgeons. However, postoperative complications such as bleeding, pain, delayed soft tissue healing, and alveolitis may occur. These complications can be recurrent and compromise the success of treatment. To reduce or prevent them, new strategies and biomaterials have been developed to improve bleeding control and tissue healing [1-3]. Recent advances in biotechnology have led to the development of absorbable topical hemostatic agents, including collagen membranes and sponges [4]. Although their precise mechanism of action is not fully understood, they are believed to act primarily through physical rather than chemical effects on the coagulation cascade [4-6]. Lumina Coat® is one of the most widely used membranes for both guided tissue regeneration and minor bleeding control. It is a malleable scaffold composed of sterilized type 1 bovine collagen. Its mode of action is well established: It forms a mechanical matrix that promotes coagulation [7,8] without interfering with the clotting cascade. Lumina Coat® is resorbed within 2-4 weeks [9,10]. Additionally, due to its hygroscopic properties [11], it absorbs blood and moisture at the application site. The membrane can retain several times its weight in fluid, a characteristic that enhances its hemostatic effect. Blood absorption concentrates platelets and clotting factors, while the resulting gel-like structure acts as a physical barrier, reducing blood loss. This dual action, combining the concentration of coagulation elements with mechanical obstruction, produces a synergistic hemostatic effect [11,12]. Hemospon® is a porcine-derived gelatin sponge (100% collagen) indicated for local hemostasis. Its mechanism of action is similar to that of Gelfoam® and other commonly used hemostatic agents in dentistry [13,14]. A previous study in rat dental alveoli [6] compared sites filled with Gelfoam® and Hemospon® and concluded that both agents produced similar biological responses in the gingival mucosa and alveolar healing. After 7 days, residual Gelfoam® was still detectable, whereas only traces of Hemospon® remained [15]. Complications related to gelatin sponges are rare and typically involve the formation of abscesses or granulomas [10,16]. Beyond their role in hemostasis, gelatin-based materials such as Hemospon® have proven useful in facilitating surgical procedures. A systematic review of hemostatic agents reported that gelatin-thrombin matrix sealants, which share similarities with Hemospon®, improve hemostatic outcomes and shorten operative times when conventional methods are insufficient [13]. The hemostatic efficacy of gelatin sponges is attributed to multiple mechanisms, particularly platelet aggregation and initiation of the coagulation cascade [17]. These properties are critical in situations requiring rapid bleeding control, as shown in studies demonstrating reduced bleeding times during surgery [18]. The well-established safety profile and versatility of gelatin sponges support their widespread use in clinical practice. A recent randomized controlled clinical study [19] compared a porcine collagen sponge (Ateloplug®) with unfilled extraction sockets (natural blood clot healing). The authors concluded that placing a collagen sponge after third molar extraction reduced early postoperative complications and improved initial soft tissue and periodontal healing [20]. The aim of this study was to evaluate and compare bleeding, soft tissue healing, and pain control following tooth extractions using either the Lumina Coat® membrane or the Hemospon® sponge. It was positively hypothesized that the collagen membrane would provide superior outcomes compared to the sponge.

## Material and Methods

This study was submitted and approved by the Human Research Ethics Committee of Centro Universitário de Santa Fé do Sul (85397324.4.0000.5428) and followed the Declaration of Helsinki (1964, updated 2024). It was designed in accordance with the CONSORT guidelines (www.consort-statement.org).

## Sample size calculation

For the design proposed, the formula used was: 


(1) $$n={\left(\frac{\left(Z_{\alpha /2}+Z_{\beta }\times \sigma \right)}{d}\right)}^{2}$$

Where n is the number of patients; σd is the standard deviation of the difference between paired observations (value is 1.35) [3]; Δ is the minimal clinically important difference (MCID) you want to detect (value is 1.08) [3]; Zα/2 is the Z-value for desired confidence (it is 1.96 for 95%); Zβ is the Z-value for desired power (for 80% power). The calculation was :


(2) $$n={\frac{\left((1.96+0.84)\times 1.35\right)}{1.08}}^{2}$$

The n resulted in 12.3 patients, rounded up to 13 patients. Therefore, adjusting for potential dropouts (considered 20%), 16 patients should be included (32 dental sockets).

## Patient selection

The selected participants were those who had two teeth requiring extraction. Each participant underwent two experimental procedures: One socket was filled with Lumina Coat® and the other with Hemospon® after extraction. Two surgeons (LLLS and FPPF) confirmed the need for extraction through clinical and radiographic examination. All research participants were informed beforehand about the physical and psychological criteria needed to participate in this study.

## Eligibility criteria

Participants in this study must meet the following clinical requirements: Indication for the extraction of two upper or lower teeth (split-mouth); older than 18 years; tooth extraction not caused by periodontal disease; no systemic disease that could affect alveolar healing or bleeding; agreement to procedures in the study; signed informed consent. Participants who do not meet these criteria were excluded from the study.

## Experimental design

**Figure 1:**
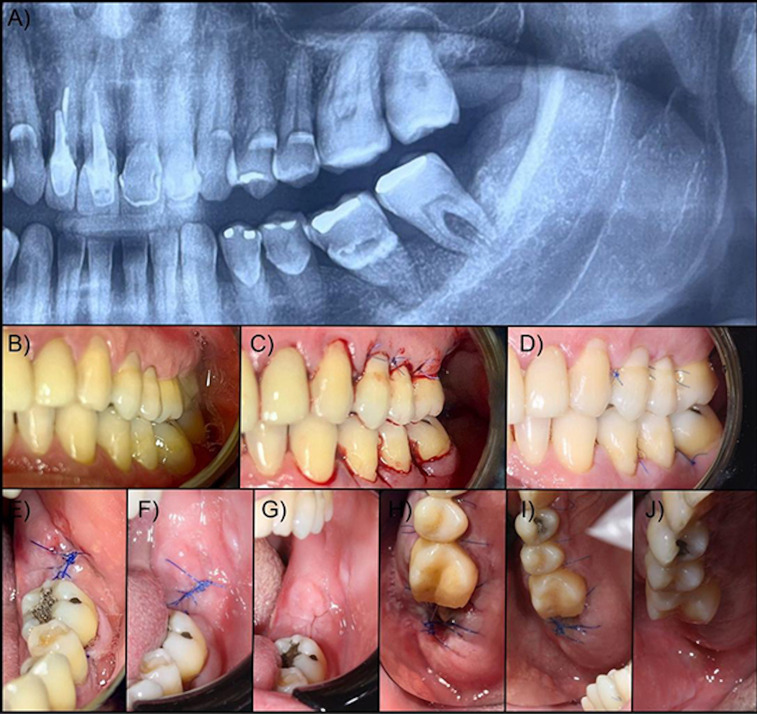
Surgical procedure performed on patients in the Lumina Coat® and Hemospon® groups. Patient 1. A: Preoperative X-ray; B: Preoperative view; C: View 30 minutes after extraction; D: View 7 days after extraction. Patient 2. E: The immediate postoperative period after extraction of the second lower molar; F: 7-day postoperative control; G: Surgical appearance after 7 days. Patient 3. H: The immediate postoperative period after extraction of the second upper molar; I: 7-day postoperative control; J: Surgical appearance after 7 days.

After the participants were selected according to the inclusion criteria, all individuals underwent a comprehensive medical history review, clinical diagnostic screening, and radiographic examination. At this stage, clinical data on oral health conditions in the region of the extracted teeth were collected using standardized intraoral photographs. All surgeries were performed by a single experienced surgeon (FPPF). All biosafety protocols were strictly followed during the surgical procedures. Following intraoral antisepsis with 0.12% chlorhexidine and extraoral antisepsis with povidone-iodine (PVPI), local anesthesia was administered using mepivacaine with adrenaline (1:100,000). Extractions were carried out using a minimally traumatic technique, including careful syndesmotomy, coronal-root sectioning, and gentle luxation with forceps and elevators. After extraction, the sockets were filled with either a collagen membrane (Lumina Coat® Critéria, São Carlos, SP, Brazil) or a Hemospon® collagen sponge. Flaps were then repositioned and closed with 5-0 polypropylene simple interrupted sutures. For postoperative care, patients received amoxicillin 500mg every 8 hours for 7 days or, in cases of penicillin allergy, azithromycin 500mg once daily for 3 days to prevent infection. Nimesulide 100mg every 12 hours for 3 days was prescribed for pain and inflammation management. Sutures were removed after 7 days. Adherence to the prescribed regimen was assessed through direct questioning during follow-up visits at 24 hours, 48 hours and 7 days postoperatively, with patients reporting on medication compliance, missed doses, and adverse reactions. In addition, all patients received both verbal and written postoperative instructions regarding medication schedules, potential side effects, and the importance of adherence. No serious adverse events or drug-related complications were reported. The step-by-step surgical protocol and follow-up schedule are shown in Figure 1.

## Blinding procedures

To minimize potential bias, the evaluator (LLLS) who conducted all postoperative assessments was blinded to the material placed in each extraction socket (Lumina Coat® or Hemospon®). Blinding was achieved by coding the materials as A and B, with allocation performed by a third party (GVOF) not involved in the evaluations. The codes were concealed from the evaluator throughout all follow-up visits. Clinical notes and patient records were redacted to remove any mention of the material used. This approach ensured that the evaluator remained unaware of group allocation at all checkpoints, maintaining the objectivity of clinical outcome assessments.

## Postoperative assessment (Clinical evaluation, bleeding, pain, and soft tissue healing)

All postoperative evaluations were carried out by the same examiner (LLLS) and always at the same time of day. Immediately after surgery, participants remained in the clinic for at least 30 minutes to monitor bleeding. They were subsequently assessed for soft tissue healing, pain, and analgesic consumption at 24 hours, 48 hours, and 7 days. Pain was assessed using the Visual Analogue Scale (VAS) [21], where 0 indicated no pain and 10 the most severe pain, complemented by a graphic rating scale. Patients also recorded and reported the number of analgesics taken. Bleeding was evaluated at 30 minutes, 24 hours, and 48 hours after suturing. It was scored from 0 to 3, where 0: No bleeding, 1: Minor bleeding on probing, 2: Immediate bleeding on probing and 3: Bleeding along the gingival sulcus with minimal stimulation, according to the Mühlemann Classification [22]. Soft tissue healing was assessed following Brancaccio et al. [23] using scores from 0 to 3. 0: Complete closure without fibrin, 1: Complete closure with fibrin, 2: Incomplete alveolar closure (dehiscence) and 3: Incomplete closure with signs of necrosis.

## Statistical analysis

Data were expressed as means and standard deviations. The D'Agostino & Pearson test was used to assess normality. Statistical analyses were performed using Prism software (v. 9, GraphPad Software). The time variable (30 minutes, 24 hours, 48 hours and 7 days) was analyzed using a non-parametric repeated-measures ANOVA, followed by the Friedman test. Pain, bleeding, and soft tissue healing were compared using the Mann-Whitney U test. A p-value ≤ 0.05 was considered statistically significant.

## Results

**Figure 2:**
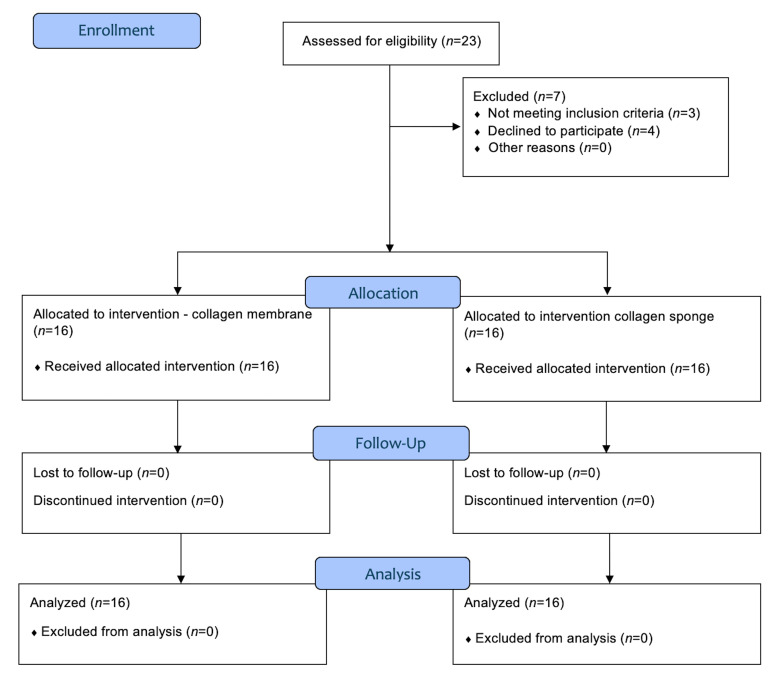
CONSORT flowchart for inclusion and follow-up of patients.

**Figure 3:**
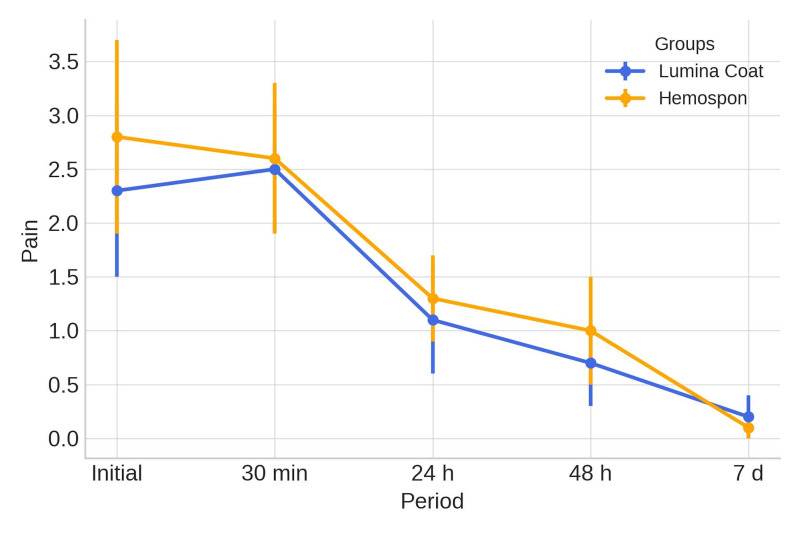
Mean and standard deviation (m±SD) of the clinical pain parameter scores. Statistical tests: Mann-Whitney (U) test (p≤0.05).

**Figure 4:**
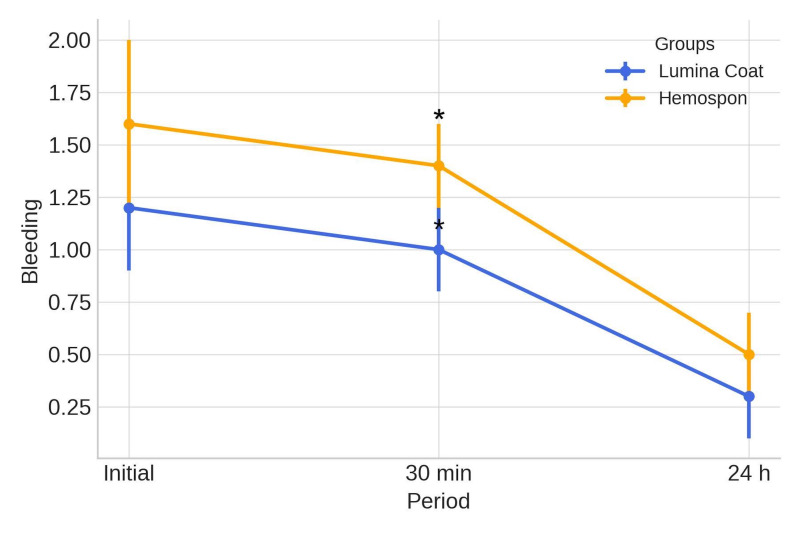
Mean and standard deviation (m±SD) of the scores for the clinical parameter of bleeding. Statistical tests: Mann-Whitney test (U). Symbols: *Statistically significant difference between the groups in the same period (p≤0.05).

Initially, twenty-three patients were invited to participate in this study; after screening, seven were excluded. A total of 16 patients were enrolled (Figure 2). The means, medians, and standard deviations (SDs) of the clinical outcomes -pain, bleeding, and soft tissue healing- are presented in Table 1 and Figures 2, 3 and 4. All participants completed the study without dropouts, and no postoperative complications were reported.

**Figure 5:**
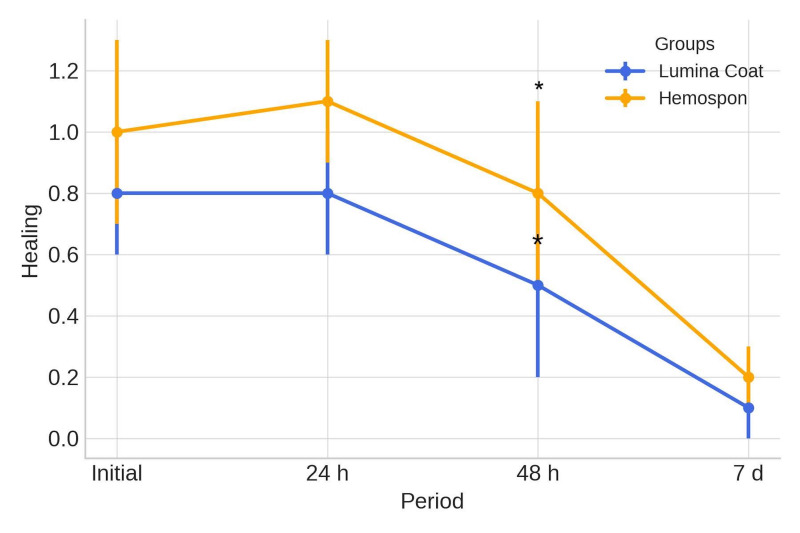
Mean and standard deviation (m±SD) of the clinical healing parameter scores. Statistical tests: Mann-Whitney test (U). Symbols: *Statistically significant difference between the groups in the same period (p≤0.05).

**Table 1:** Results of the comparisons between times and groups of the clinical parameters pain, bleeding and healing.

Clinical Parameter	Time	Lumina Coat Mean ± SD	Lumina Coat Median ± IIQ	Hemospon Mean ± SD	Hemospon Median ± IIQ	p-value *
Pain	Start	2.20 ± 1.58 a	2.00 ± 2.00	2.80 ± 1.77 a	3.00 ± 2.25	0.257
Pain	30 min	2.60 ± 1.43 a	2.00 ± 2.25	2.60 ± 1.43 a	2.00 ± 2.25	1.000
Pain	24 hours	1.05 ± 0.95 b	1.00 ± 2.00	1.20 ± 1.11 b	1.00 ± 2.00	0.724
Pain	48 hours	0.40 ± 0.60 c	0.00 ± 1.00	0.75 ± 0.91 bc	0.50 ± 1.00	0.233
Pain	7 days	0.25 ± 0.44 c	0.00 ± 0.25	0.29 ± 0.31 c	0.00 ± 0.22	0.722
Bleeding	Start	1.35 ± 0.99 a	1.00 ± 1.00	1.65 ± 0.88 a	1.5 ± 1.00	0.249
Bleeding	30 min	0.75 ± 0.44 b	1.00 ± 0.25	1.20 ± 0.62 b	1.00 ± 1.00	0.015
Bleeding	24 hours	0.20 ± 0.41 b	0.00 ± 0.00	0.40 ± 0.60 c	0.00 ± 1.00	0.273
Bleeding	48 hours	0.00 ± 0.00 c	0.00 ± 0.00	0.00 ± 0.00 d	0.00 ± 0.00	1.000
Bleeding	7 days	0.00 ± 0.00 c	0.00 ± 0.00	0.00 ± 0.00 d	0.00 ± 0.00	1.000
Healing	Start	0.80 ± 0.83 a	1.00 ± 1.25	1.00 ± 0.80 ab	1.00 ± 2.00	0.429
Healing	1 day	0.80 ± 0.83 a	1.00 ± 1.25	1.15 ± 0.75 a	1.00 ± 1.00	0.168
Healing	2 days	0.35 ± 0.49 b	0.00 ± 1.00	0.80 ± 0.70 b	1.00 ± 1.00	0.034
Healing	7 days	0.00 ± 0.00 c	0.00 ± 0.00	0.05 ± 0.22 c	0.00 ± 0.00	0.342

The times were compared using non-parametric repeated measures ANOVA using the Friedman test (p<0.0001 for the times of each parameter in each group). Different superscript letters indicate differences in means/medians between times. * Comparisons between 2 groups were made using the Mann-Whitney U-test.

### Pain

In the later postoperative period, pain scores decreased in both groups. Baseline pain scores were 2.20 for the Lumina Coat group and 2.80 for the Hemospon group. By day 7, both groups had an average score of 0.25. No statistically significant differences were observed between the groups at any time point. The means and standard deviations of the pain scores are shown in Table 1 and Figure 3.

### Bleeding

Bleeding scores were higher in both groups in the early postoperative phase. The Lumina Coat group had an average score of 1.35, while the Hemospon group averaged 1.65. Scores progressively declined over the first 24 hours, after which bleeding ceased completely. A statistically significant difference was observed at 30 minutes postoperatively, with the Lumina Coat group showing lower scores (average 0.75) compared to the Hemospon group (average 1.20). The means and standard deviations of the bleeding scores are shown in Table 1 and Figure 4.

### Healing

Healing scores were higher in both groups during the early postoperative period but decreased steadily until day 7. At 2 days postoperatively, a statistically significant difference was observed, with the Lumina Coat group averaging 0.35 compared to 0.80 in the Hemospon group. The means and standard deviations of the healing scores are shown in Table 1 and Figure 5.

## Discussion

The use of membranes in dentistry, particularly in periodontics and oral surgery, has broad applications, including socket preservation after extraction [3] and guided tissue or bone regeneration [24-26]. Despite these benefits, post-extraction complications such as periodontal tissue recession, trismus, pain, swelling [27,28], and suture dehiscence remain significant challenges. This study investigated collagen membranes as an alternative to collagen sponges, aiming to reduce postoperative morbidity and enhance patient comfort. Collagen membranes demonstrated the ability to reduce initial bleeding and accelerate periodontal soft tissue healing. Collagen is highly biocompatible, elicits minimal tissue reaction, and has low immunogenicity [29]. However, exposure to oral bacteria raises concerns about early degradation and infection risk [30]. Functionally, collagen promotes wound stabilization, hemostasis, and periodontal ligament cells and gingival fibroblast chemotaxis, reinforcing its value in clinical practice. Collagen sponges are widely used to support alveolar healing, protect palatal donor sites, and control intraoperative bleeding [31]. The properties of collagen membranes vary with thickness and structure, which influence cell migration and tissue integration. Thicker membranes provide greater restriction of soft tissue cells' migration, strengthening their role in guided tissue regeneration (GTR) [32]. An ideal membrane balances compressive strength with clinical flexibility, serving not only as a barrier but also as an active matrix for regenerative processes mediated by osteoblasts and other cells [33]. Collagen-based products may also improve postoperative comfort. Some evidence indicates reduced pain compared with sites where no collagen was used [34], though results are limited by the lack of negative control groups in several studies [35]. Collagen sponges similarly reduce pain in extraction sockets and donor sites [36]. In the present study, pain scores did not differ significantly between Lumina Coat® and Hemospon®. A recent randomized, double-blind split-mouth trial involving 26 patients compared a novel gelatin-based sponge with Gelfoam/Spongostan® in mandibular molar extractions. The test sponge provided superior bleeding control at 1, 4, and 24 hours (p<0.05), reduced pain up to 48 hours, and showed fewer dry socket cases [37]. Likewise, Maffei et al. [3] reported lower postoperative pain with collagen membranes than with soft tissue grafts. Hemostasis in oral surgery typically relies on sutures, vasoconstrictors, and careful planning for patients with clotting disorders [38]. Nevertheless, some patients present persistent bleeding [39]. Collagen enhances hemostasis through platelet activation and aggregation [40]. In this study, Lumina Coat achieved lower bleeding scores than Hemospon after 30 minutes, consistent with previous reports [35,44]. A retrospective study of 200 patients (741 extraction sites) found that collagen placement minimized postoperative bleeding in anticoagulated patients, with only eight minor bleeding events, all in anti-vitamin K users [41]. Collagen membranes also help preserve soft tissue architecture, preventing epithelial invasion of the alveolus and promoting selective cell colonization. This enhances graft integration, reduces infection risk, and supports better healing outcomes [42]. Favorable results with collagen biomaterials in soft tissue thickness and dimensional maintenance after extraction are well documented [36]. Collagen degradation dynamics involve metalloproteinases (MMPs) secreted by macrophages and fibroblasts, which influence the membrane's barrier capacity and could lead to increased inflammation or early exposure to the oral environment. Proper management of the collagen exposure is important because it can accelerate degradation and increase inflammatory tissue reactions [43]. The lack of early membrane exposure in this study emphasizes the importance of proper handling and application techniques, which are essential for maximizing the clinical success of collagen-based treatments. Hemostasis caused by collagen results from the interactions and activation of platelets, as well as the positive modulation of their adhesion and aggregation. In the present study, using Lumina Coat resulted in a lower bleeding score after 30 minutes compared to Hemospon. In a study [44], the use of collagen membranes in wounds that healed by second intention prevented total postoperative bleeding in 80% of cases. The remainder, although experiencing slight bleeding, did not require any hemostatic maneuvers. A collagen sponge or membrane acts as an extracellular matrix, enhancing coagulum revascularization and fibroblast activity and promoting alveolar repair. It prevents the soft tissue from collapsing, which blocks the invasion of epithelial cells. Maintaining the space allows colonizing cells, such as bone cells, and also prevents food from entering the healing alveolus, which could lead to infection. In the present study, using Lumina Coat produced results consistent with those reviewed by Bunyaratavej and Wang [45], Sbricoli et al. [46], and Mizraji et al. [47], which showed significant improvement in periodontal healing. The positive impact of collagen biomaterials was also seen in helping to maintain the thickness and size of the soft tissue when used in post-extraction sockets. During the breakdown of the fibrous skeleton formed after clot removal, collagen is degraded by MMPs released by macrophages, neutrophils, and fibroblasts. Early degradation of the collagen barrier can reduce its ability to act as a barrier, and bacterial contamination of the alveolus after extraction can worsen the clinical outcomes. In some instances, unintentional exposure of the collagen membrane in the oral cavity accelerates its degradation, which is linked to a heightened inflammatory response in nearby tissue. In this study, no patients were exposed to the membrane or collagen sponge before the study started. Most published studies on collagen membranes focus on ridge/socket preservation and regenerative outcomes, rather than pain, bleeding, or soft tissue healing. The present study addresses this gap, though limitations should be noted: Variable patient adherence to prescribed medications, a relatively short follow-up period, and the lack of evaluation of membrane-sponge combinations or biomodifications with growth factors. These avenues warrant further clinical investigation.

## Conclusions

Both Lumina Coat (membrane) and Hemospon (sponge) proved effective for controlling bleeding, reducing pain, and promoting soft tissue healing. While the null hypothesis was not rejected, Lumina Coat showed superior outcomes in bleeding reduction and healing scores. Clinically, minimizing bleeding and accelerating soft tissue repair are critical considerations when selecting biomaterials for post-extraction management.
